# Relationship between resilience and self-efficacy among Iranian nurses: a cross-sectional study during the post-Corona era

**DOI:** 10.1186/s12912-024-01894-0

**Published:** 2024-04-16

**Authors:** Saeed Ghasempour, Ali Abbasi, Mohammad Hasan Basirinezhad, Ali Dadgari, Hossein Ebrahimi

**Affiliations:** 1 Student Research Committee, School of Nursing and Midwifery, Shahroud University of Medical Sciences, Shahroud, Iran; 2 Department of Nursing, School of Nursing and Midwifery, Shahroud University of Medical Sciences, Shahroud, Iran; 3 Department of Epidemiology and Biostatistics, School of Public Health, Shahid Sadoughi University of Medical Sciences, Yazd, Iran; 4 Center for Health Related Social and Behavioral Sciences Research, Shahroud University of Medical Sciences, Shahroud, Iran

**Keywords:** Nurses, Resilience, Self-efficacy, COVID-19

## Abstract

**Background:**

Resilience and self-efficacy play an influential role in nurses’ clinical performance, which are considered resources for improving adaptability and promoting work engagement. This study aimed to determine the relationship between resilience and self-efficacy among nurses at Shahroud University of Medical Sciences hospitals during the post-Corona era.

**Methods:**

This cross-sectional study was conducted on 280 nurses in all clinical departments. Nurses with a bachelor of science in nursing or higher degree and at least one year of full-time work experience were included in the study using a convenience sampling method. Participants completed a three-part tool, which included the demographic information form, the Connor-Davidson Resilience Scale, and the General Self-Efficacy Scale. The data were analyzed using descriptive statistics and inferential tests (multivariate linear regression using the backward method).

**Results:**

In this study, nurses reported low levels of resilience (63.64 ± 15.66) and high levels of self-efficacy (63.01 ± 9.57). Among the five resilience subscales, the highest mean item score was associated with “spiritual influences” (2.80 out of 4), while the lowest mean item score was associated with “trust in one’s instincts and tolerance of negative affect” (2.36 out of 4). Furthermore, the multivariate linear regression model results indicated that self-efficacy accounted for 33.6% of the variance in resilience (*P <* 0.001 and *β =* 0.952).

**Conclusion:**

According to the results of the present study, it is suggested that nurses’ psychological capabilities, such as self-efficacy, should be increased to improve resilience and address the stressful conditions of the work environment.

## Introduction

Nurses play a crucial role in patient care as one of the most important pillars of the healthcare system [1]. The challenges nurses face include workplace conditions, long working hours, caring for sick or dying patients, and quantitative and qualitative work pressure [2]. Furthermore, the COVID-19 pandemic has presented nurses with additional challenges, including insufficient knowledge about the effects of the Coronavirus, unprepared caring protocols for patients with it, inadequate personal protective equipment (PPE), and concerns about their families and children [3, 4]. As a result, nurses have experienced various problems, such as burnout [5, 6], stress, anxiety, and depression [7, 8]. Nurses at the frontline of the fight against this pandemic have been more closely connected to this emerging virus and its sufferers than other groups in society, which affected different aspects of their lives. Therefore, it is crucial to identify factors that can assist nurses in better dealing with critical and unpredictable situations such as COVID-19 [9, 10].

Resilience is one of these factors that expresses a person’s capacity to adapt positively and successfully to difficult and unfortunate life conditions [11], which facilitates interactions between a person and their environment [12]. This complex and dynamic process enables nurses to positively adapt to workplace stressors, cope with psychological trauma, and ultimately provide safe and high-quality nursing care to patients [13, 14]. Resilience plays a crucial role in the adaptation and effective treatment of healthcare workers (HCWs), particularly nurses, during the COVID-19 pandemic. It acts as a protective shield against psychological issues like stress and depression [15]. It is important to note that understanding nurses’ resilience and identifying its related factors is crucial for maintaining their well-being, enhancing their performance, and ensuring the delivery of high-quality nursing care [16].

Based on the literature review, it seems that self-efficacy is one of the factors related to nurses’ resilience [10, 17]. This factor reflects a person’s beliefs about their ability to perform behaviors with certain consequences [18]. Self-efficacy theory assumes that people’s beliefs about their skills and talents positively impact their actions and serve as the foundation for their activities [19]. This psychological concept holds particular significance in the nursing profession. The term refers to the general competence of nurses in carrying out their duties, which encompasses acquiring clinical knowledge and skills, effective communication with patients, and adherence to nursing ethics [20, 21]. Various studies have shown that nurses with higher self-efficacy perform better and provide higher quality care than nurses with lower self-efficacy. These nurses are more committed to their work and demonstrate greater endurance when facing problems [22–24]. Conversely, nurses with lower self-efficacy may delay providing care or even avoid it altogether, resulting in harm to patients [25].

It should be noted that several studies were conducted on the mental health of HCWs, especially nurses, around the world during the COVID-19 pandemic. However, with the announcement of the end of this pandemic by the World Health Organization (WHO), researchers’ focus on the psychological issues of nurses has decreased. Thus, there are limited studies on the relationship between psychological variables such as resilience and self-efficacy in the post-Corona era, and many research gaps are evident on this issue. On the other hand, these two variables play a crucial role in nurses’ clinical performance, and it appears that understanding the relationship between them will lead to providing suitable solutions and approaches to enhance mental health and improve the clinical performance of nursing personnel. Therefore, since there was no study to determine the relationship between these two variables in the post-Corona era, this study was performed to determine the relationship between resilience and self-efficacy among nurses at Shahroud University of Medical Sciences hospitals during the post-Corona era.

## Materials and methods

### Study design and participants

This cross-sectional study was conducted from June to December 2022 to investigate the relationship between resilience and self-efficacy among nurses at Shahroud University of Medical Sciences hospitals. Two hundred eighty nurses working in all clinical departments of Imam Hossein (PBUH) and Bahar hospitals were included in the study based on the inclusion and exclusion criteria through the convenience sampling method.

The inclusion criteria were as follows: (1) having a bachelor of science in nursing (BSN) or a higher degree and (2) having at least one year of full-time work experience in a hospital setting. The exclusion criteria also included: (1) transferring between departments within the last month and (2) having any chronic disease. Chronic diseases considered important in this study included cancer, arthritis, chronic obstructive pulmonary disease (COPD), diabetes, cardiovascular disease (CVD), hypertension, obesity, osteoporosis, and stroke, which were expressed based on self-reports from nurses.

The sample size was estimated to be 280 nurses based on Shahrbabaki et al. (2023) [26], considering the power of 80% at the confidence level of 99% and including a 5% dropout of samples.

Type I error (**α**) = **0.01** Type II error (**β**) = **0.20** Correlation coefficient (**r**) = **0.21**

$$n = {\left[ {\frac{{{Z_{1 - {\raise0.5ex\hbox{$\scriptstyle \alpha $}\kern-0.1em/\kern-0.15em\lower0.25ex\hbox{$\scriptstyle 2$}}}} + {Z_{1 - \beta }}}}{{\frac{1}{2}\log \frac{{1 + r}}{{1 - r}}}}} \right]^2} + 3 = 260$$

### Data collection tools

The data collection tool in this study consisted of three parts distributed among nurses in each department who were asked to complete the questionnaires in their free time.

#### Part 1. Demographic information form

This form included information about age, gender, marital status, education level, work experience, work experience in the COVID-19 department, income adequacy, department of work, and satisfaction with the PPE of nurses.

#### Part 2. Connor-Davidson Resilience Scale (CD-RISC)

The Connor-Davidson Resilience Scale was designed by Connor and Davidson in 2003. This scale consists of 25 items that assess five components: personal competence (items 25, 24, 23, 17, 16, 12, 11, and 10), trust in one’s instincts, tolerance of negative affect (items 20, 19, 18, 15, 14, 7, and 6), positive acceptance of change and secure relationships (items 8, 5, 4, 2, and 1), control (items 22, 21, and 13), and spiritual influences (items 9 and 3). Items are scored based on a five-point Likert scale (not true at all = 0, rarely true = 1, sometimes true = 2, often true = 3, and true nearly all the time = 4) [27]. Therefore, the scores range from 0 to 100, with scores of 0 to 65 indicating low resilience, 66 to 79 indicating moderate resilience, and 80 to 100 indicating high resilience [28]. Connor and Davidson (2003) reported the reliability of their scale using internal consistency, with a Cronbach’s alpha of 0.87. They also assessed its validity through factor analysis, as well as convergent and divergent validity in different groups, including normal and at-risk individuals. The results confirmed the scale’s acceptable validity and reliability [27]. In a study by Bakhshayesh Eghbali et al. (2022), the psychometrics of the Persian version of this scale were assessed. The confirmatory factor analysis (CFA) results for the five components mentioned showed that 25 scale items had high factor loadings and favorable fit indices. Therefore, the CD-RISC demonstrates good construct validity. Cronbach’s alpha coefficients for the entire scale and its subscales, including personal competence, trust in one’s instincts, tolerance of negative affect, positive acceptance of change and secure relationships, control, and spiritual influences, were calculated as 0.94, 0.89, 0.80, 0.71, 0.77, and 0.74, respectively, indicating good reliability of the CD-RISC in Iranian society [29]. In the present study, the reliability of the Persian version of this scale was also calculated using Cronbach’s alpha method, 0.94.

#### Part 3. General Self-Efficacy Scale (GSES)

The General Self-Efficacy Scale was developed by Sherer et al. (1982) and measures a person’s beliefs about their ability to overcome various situations. This scale has 17 items scored on a five-point Likert scale ranging from 1 (strongly disagree) to 5 (strongly agree). It is worth noting that items 2, 4, 5, 6, 7, 10, 11, 12, 14, 16, and 17 are reverse scored. In other words, a score of 5 is given to “strongly disagree”, and a score of 1 is given to “strongly agree” [30]. The minimum and maximum scores obtained on this scale are 17 and 85, respectively. Scores ranging from 17 to 28 indicate low self-efficacy, 29 to 57 indicate moderate self-efficacy, and 58 to 85 indicate high self-efficacy [31]. Sherer et al. (1982) calculated the reliability coefficient of this scale using a Cronbach’s alpha method of 0.86. They also examined its construct and criterion validity. Thus, a significant negative correlation was found between the GSES score and the Rotter’s Locus of Control Scale (RLCS) score. Furthermore, there was a significant positive correlation between the GSES score and the Social Desirability Scale of Marlow and Crown (SDS) score [30]. Asgarnezhad et al. (2006) conducted a psychometric study on the Persian version of this scale, and the results confirmed its validity and reliability. They used exploratory and confirmatory factor analysis methods to examine construct validity. The exploratory factor analysis (EFA) revealed three factors: willingness to initiate behavior, willingness to expend effort to complete the behavior, and persistence in the face of adversity. The results of CFA supported a three-factor model with a higher-order factor (self-efficacy) [32–34]. Furthermore, the reliability of the Persian version of this scale was calculated using Cronbach’s alpha coefficient, 0.83 [32]. In the present study, the reliability of this scale was obtained by Cronbach’s alpha method, 0.87.

### Ethical considerations

After obtaining the necessary permits from the Vice President of Research and Technology and the Research Ethics Council of Shahroud University of Medical Sciences (Ethics code: IR.SHMU.REC.1400.267), necessary arrangements were made with the officials of Imam Hossein (PBUH) and Bahar hospitals. Subsequently, the study’s objectives were explained to all participating nurses, and their verbal and written informed consent was obtained for participation in the study.

### Statistical analysis

The data were analyzed using descriptive statistics (frequency, percentage, mean, and standard deviation) and inferential tests (multivariate linear regression using the backward method) in Statistical Package for the Social Sciences (SPSS) version 16 software. A significance level of 0.05 was considered for all tests.

## Results

In this study, the majority of participants were female (86.8%) and married (73.9%). The participants’ mean and standard deviation of age and work experience were 32.53 ± 6.01 and 8.94 ± 5.77, respectively. The other demographic characteristics of the participating nurses are given in Table 1. In addition, the participants’ mean scores of resilience and self-efficacy were reported as 63.64 ± 15.66 and 63.01 ± 9.57, respectively. The mean resilience scores of the participating nurses according to the subscales are given in Table 2.

**Table 1:** The demographic characteristics of participating nurses (*N* = 280)

Variables		*N*	%
**Gender**	Male	37	13.2
	Female	243	86.8
**Marital status**	Married	207	73.9
	Single	73	26.1
**Education level**	BSN	267	95.4
	MSN	13	4.6
**Income adequacy**	More than enough	25	8.9
	Just enough	81	28.9
	Less than enough	174	62.2
**Department of work**	Internal	49	17.5
	Surgery	39	13.9
	Neonatal and pediatric	17	6.1
	Maternity	28	10.0
	Psychiatry	5	1.8
	ICU	84	30.0
	Emergency Room	39	13.9
	Operating Room	19	6.8
**Satisfaction with PPE**	Not satisfied	69	24.6
	Somewhat satisfied	87	31.1
	Satisfied	124	44.3
		**Mean**	**SD**
**Age (year)**		32.53	6.01
**Work experience (year)**		8.94	5.77
**Work experience in the COVID-19 department (month)**		6.38	10.50

**N**: Frequency; **%**: Percent; **BSN**: Bachelor of Science in Nursing; **MSN**: Master of Science in Nursing; **ICU**: Intensive Care Unit; **PPE**: Personal Protective Equipment; **SD**: Standard Deviation

**Table 2:** The mean score of resilience and its subscales of participating nurses

Variables		Min (obtainable)	Max (obtainable)	Mean	SD
**Resilience**	**Total**	0	100	63.64	15.66
	Personal competence	0	32	20.30	5.82
	Trust in one’s instincts, tolerance of negative affect	0	28	16.54	4.81
	Positive acceptance of change and secure relationships	0	20	13.62	3.23
	Control	0	12	7.58	2.34
	Spiritual influences	0	8	5.60	1.74

**Min**: Minimum; **Max**: Maximum; **SD**: Standard Deviation

**Fig. 1:**
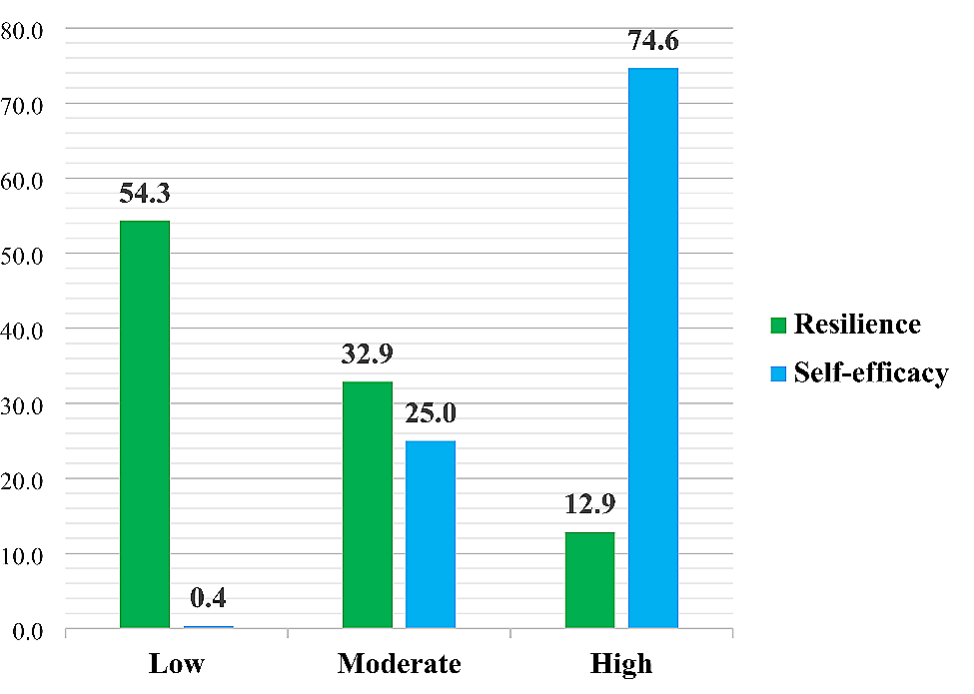
The level of resilience and self-efficacy of participating nurses

**Fig. 2:**
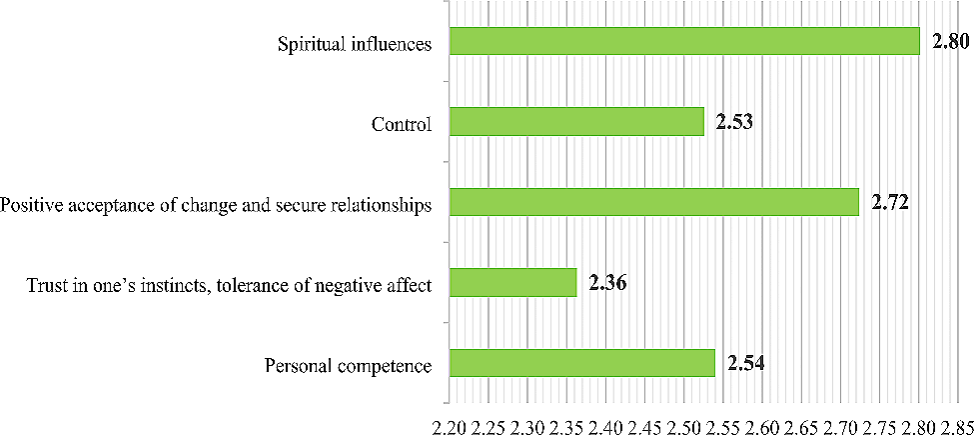
Comparison of the mean scores of all five resilience subscales

Overall, 54.3% of nurses experienced low levels of resilience, while 74.6% reported high levels of self-efficacy. The different levels of resilience and self-efficacy of the participating nurses are shown in Fig. 1. A comparison of mean item scores of five resilience subscales showed that the highest and lowest mean item scores corresponded to the subscales of “spiritual influences” (2.80 out of 4) and “trust in one’s instincts and tolerance of negative affect” (2.36 out of 4), respectively. The comparison of the mean item scores of all five resilience subscales is shown in Fig. 2.

The regression model was first analyzed via univariate analysis. Variables with a significance level of less than 0.2 were subsequently entered into the multivariate model. Finally, the multivariate linear regression model was implemented using the backward method. The results of this model indicate that 33.6% of the variance in resilience is explained by self-efficacy. As shown in Table 3, for each unit increase in self-efficacy, the mean resilience score increased by 0.952 units (*P <* 0.001).

**Table 3:** The role of independent variables on resilience based on a multivariate linear regression model

Model	β	SE	t	*P*
**(Constant)**	3.666	5.092	0.720	0.472
**Self-efficacy**	0.952	0.080	11.911	< 0.001

**SE**: Standard Error; **P**: P-value

## Discussion

The present study was conducted with the aim of determining the relationship between resilience and self-efficacy among nurses at Shahroud University of Medical Sciences hospitals during the post-Corona era. According to the results, nurses in the post-Corona era obtained a mean resilience score of 63.64 ± 15.66. This figure was reported as 64.94 ± 21.53 in the study by Li et al. (2023), which examined factors related to nurses’ self-efficacy two years after the COVID-19 outbreak in Wuhan, China, the origin of this virus. This finding aligns with the results of the current study [35]. Among other important findings of the mentioned study, it is worth noting the negative and significant correlation of self-efficacy with depression and anxiety, as well as its positive and significant correlation with resilience [35]. Additionally, the findings revealed that 54.3% of nurses exhibited low resilience during this period. This contrasts with the study conducted by Pachi et al. (2024), which focused on insomnia, nightmares, and their association with the mental resilience of nurses in the post-Corona era. In their study, only 24.5% of Greek nurses demonstrated low resilience [36]. This difference may be attributed to variations in the measurement tools used to assess nurses’ resilience. The mentioned study utilized the Brief Resilience Scale (BRS), which has different scoring and cut-off points compared to the CD-RISC. Despite the contradiction obtained, what is important is the adverse effects of this virus on the resilience of HCWs, especially nurses. These effects can still be seen in the post-Corona era. This highlights the need to use appropriate solutions and approaches to improve resilience and reduce the negative effects of this pandemic on the mental health of nurses.

In this study, among the five resilience subscales, the highest mean item scores were related to the subscale of spiritual influences. In line with the above finding, in the study of Alameddine et al. (2021), which was conducted on nurses working in referral centers for the hospitalization of patients with COVID-19 in Lebanon, the highest mean item scores were also reported for this subscale [37]. This topic highlights the important role of spirituality and religion among Middle Eastern nurses. According to Weathers (2018), religion, spirituality, and personal beliefs serve as sources of strength that can help nurses adapt better to stressful work and life conditions and increase their resilience against problems [38]. Therefore, nurses’ spiritual outlook plays a key role in dealing with potentially stressful and sudden situations such as COVID-19 [37]. In addition, the lowest mean item scores were related to the subscale of trust in one’s instincts and tolerance of negative affect. This finding aligns with the results of two previous studies by Alameddine et al. (2021) [37] and Shahrbabaki et al. (2023) [26], which also reported the lowest mean item scores for this subscale. Alameddine et al. (2021) considered the low mean item scores in two subscales of trust in one’s instincts, tolerance of negative affect, and personal competence to be caused by the low self-efficacy of nurses [37]. However, in this study, despite the low mean item scores on these two subscales, 74.6% of nurses experienced high levels of self-efficacy. This discrepancy may be due to the absence of a specific tool to measure nurses’ self-efficacy in the mentioned study, as well as socio-cultural conditions governing societies and the different life patterns of the people.

Additionally, a positive and significant relationship was found between nurses’ resilience and self-efficacy. Various studies have also listed self-efficacy as one of the factors associated with nurses’ resilience [10, 17, 35, 39, 40]. It should be noted that self-efficacy is a key personal resource that is related to self-directed motivation, positive expectations of success, and a greater capacity to resist challenging work situations. This concept predicts positive states such as the ability to adapt successfully or improve adverse conditions, which can help enhance the performance of HCWs in difficult and exhausting work environments [17]. In this regard, Cabrera-Aguilar et al. (2023) mentioned self-efficacy and resilience as essential resources for improving adaptability and promoting nurses’ work engagement [17]. According to Guo et al. (2017), nurses with high self-efficacy view problems as opportunities for improvement rather than threats to be avoided. Self-efficacy helps nurses effectively cope with clinical challenges, leading to improved resilience and successful adaptation to demanding work environments [10]. The significance of this issue lies in the fact that resilience is considered a crucial factor in managing stressful situations and preventing emotional exhaustion, mental fatigue, lack of motivation, and ultimately intention to leave this profession [10, 17]. Thus, understanding the relationship between these two variables is essential in creating a satisfactory work environment and retaining nurse professionals.

### Research limitations and recommendations

The most important limitation of this study is the use of self-reporting tools, which may not have answered the questions responsibly and correctly. In addition, this study was only conducted on nurses working at Shahroud University of Medical Sciences hospitals, which makes it difficult to generalize the results to medical centers at other medical sciences universities in the country and non-university centers, such as private medical centers. Therefore, it is suggested that similar future studies should be conducted with a larger sample size.

## Conclusion

In this study, nurses reported low resilience and high self-efficacy levels. Additionally, a positive and significant relationship was observed between nurses’ resilience and self-efficacy. Therefore, nursing managers play a crucial role in positively adapting to challenging workplace conditions and handling unpredictable situations such as COVID-19 by holding resilience skills training workshops and increasing the psychological capabilities of nurses, including self-efficacy.

## Data Availability

The datasets used and/or analyzed during the current study are available from the corresponding author upon reasonable request.

## Abbreviations

PPE: Personal Protective Equipment
HCWs: Healthcare Workers
WHO: World Health Organization
PBUH: Peace Be Upon Him
BSN: Bachelor of Science in Nursing
COPD: Chronic Obstructive Pulmonary Disease
CVD: Cardiovascular Disease
CD-RISC: Connor-Davidson Resilience Scale
CFA: Confirmatory Factor Analysis
GSES: General Self-Efficacy Scale
RLCS: Rotter’s Locus of Control Scale
SDS: Social Desirability Scale
EFA: Exploratory Factor Analysis
SPSS: Statistical Package for the Social Sciences
BRS: Brief Resilience Scale
